# Phospholipid transport protein function at organelle contact sites

**DOI:** 10.1016/j.ceb.2018.04.011

**Published:** 2018-08

**Authors:** Shamshad Cockcroft, Padinjat Raghu

**Affiliations:** 1 Department of Neuroscience, Physiology and Pharmacology, Division of Biosciences, University College London, London WC1E 6JJ, UK; 2 National Centre for Biological Sciences, TIFR-GKVK Campus, Bellary Road, Bangalore 560065, India

## Abstract

Phospholipids are synthesized at the endoplasmic reticulum (ER), the largest membrane bound organelle that forms membrane contact sites (MCS) with almost every other organelle. MCS are locations at which the membrane

es of two organelles are closely positioned to provide a microenvironment where proteins in one membrane can interact with the opposite membrane. Thus, MCS provide an ideal location at which lipid transfer proteins (LTPs) can achieve the efficient transfer of individual classes of lipids from the ER to other organelles via non-vesicular transport. Here we provide an overview of emerging findings on the localization and biochemical activity of LTPs at MCS between the ER and other cellular membranes. The localization of LTPs at MCS offers an elegant cell biological solution to tune local lipid composition to ongoing cell physiology.

**Current Opinion in Cell Biology** 2018, **53**:52–60

This review comes from a themed issue on **Membrane trafficking**

Edited by **Anne Spang** and **Satyajit Mayor**

For a complete overview see the Issue and the Editorial

Available online 30 May 2018

**https://doi.org/10.1016/j.ceb.2018.04.011**

0955-0674/© 2018 The Authors. Published by Elsevier Ltd. This is an open access article under the CC BY license (http://creativecommons.org/licenses/by/4.0/).

## Introduction

**Figure 1:**
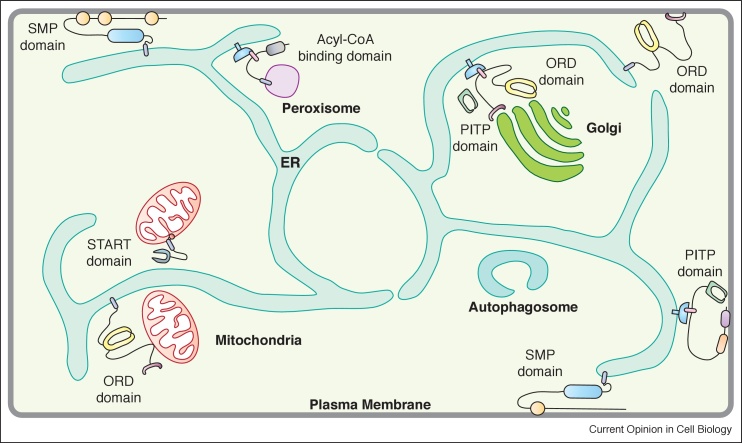
Lipid transfer at membrane contact sites. The ER is the main site of lipid synthesis and makes contact with many organelles. At these membrane contact sites, lipid transfer proteins from different families defined by the presence of specific domains such as the ORD, START, Acyl-CoA, SMP and PITP domains mediate lipid exchange. *Abbreviations*: ORD, OSBP-related domain; START, StAR-related lipid-transfer; SMP, synaptotagmin-like mitochondrial; PITP, phosphatidylinositol transfer protein domain.

The endoplasmic reticulum (ER) is the main site of phospholipid synthesis and provides lipids to other membrane compartments by vesicular and non-vesicular transport. Non-vesicular transport relies on lipid transfer proteins (LTPs) that can move lipids between membranes through aqueous cytosol. The ER is an elaborate network of membranes making contact with nearly all organelles including mitochondria, plasma membranes (PM), endosomes, lysosomes, peroxisomes, Golgi apparatus, lipid droplets and autophagosomes (Figure 1). These areas of close contact, referred to as membrane contact sites (MCS), are formed by transient associations or can be stably present depending on cell type and context. The gap between two membranes at MCS is generally 10–30 nm spanned by tethering proteins. One of the many functions of MCS is the transfer of lipids by LTPs. LTPs are distinguished by the presence of domains such as the START (StaR related lipid-transfer), ORD (*O*SBP-*r*elated *d*omain), Acyl-CoA, SMP (*s*ynaptotagmin-like *m*itochondrial-lipid binding *p*rotein) and PITP (*p*hosphatidyl*i*nositol *t*ransfer *p*rotein) domains (Figure 1). Most LTP domains contain hydrophobic cavities that can accommodate a single lipid and are highly selective. LTPs fall into two categories: single domain proteins constituted of solely the lipid binding domain and multi-domain proteins where an LTP domain is associated with additional domains [1,2] (see Figure 3 for examples).

The purpose of this review is to discuss emerging concepts of how lipid transfer between membrane compartments is facilitated at these MCS. In depth reviews on LTPs and MCS can be consulted for background information [2, 3, 4, 5].

## Lipid exchange at ER–PM contact sites

**Figure 2:**
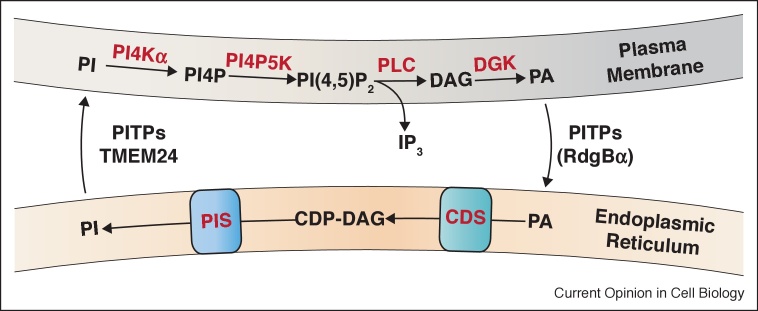
Transfer of PI and PA during the PI(4,5)P_2_ cycle triggered by PLC activation. The enzymes are distributed between two membrane compartments, the ER and PM. Lipid transfer between these compartments is required at two points in the cycle; transfer of PI from the ER to the plasma membrane and transfer of phosphatidic acid (PA) from the plasma membrane to the ER. *Abbreviations*: PIS, PI synthase; CDS, CDP-diacylglycerol synthase; DGK, diacylglycerol kinase; PI4K, PI 4-kinase; PIP5K, PI4P 5-kinase; PLC, phospholipase C; PA, phosphatidic acid; DAG, diacylglycerol.

The PM of cells has a unique lipid composition being enriched in phosphoinositides and phosphatidylserine. Phosphoinositides are low abundance lipids generated by the phosphorylation of the precursor lipid phosphatidylinositol (PI) which is synthesized in the ER (Figure 2). The two most abundant phosphoinositides, phosphatidylinositol(4,5)bisphosphate (PI(4,5)P_2_) and its precursor phosphatidylinositol 4-phosphate (PI4P) are enriched in the inner leaflet of the PM where they serve many functions including regulation of the actin cytoskeleton, ion channel activity and exo-endocytosis. In addition, receptor-regulated phospholipase C (PLC) hydrolyses PI(4,5)P_2_ to generate the second messengers, inositol(1,4,5)trisphosphate (IP_3_) and diacylglycerol (DAG). During PLC signaling, PI(4,5)P_2_ levels can drop rapidly at the PM requiring compensatory resynthesis to ensure stable levels of this key lipid. The biochemical pathway triggered by PI(4,5)P_2_ hydrolysis and leading to its resynthesis includes five lipid intermediates that are distributed between the ER and the PM (PI(4,5)P_2_ cycle); this leads to a topological constraint requiring transfer of lipid intermediates between the ER and PM [4] (Figure 2).

### Phosphatidylinositol transfer proteins

**Figure 3:**
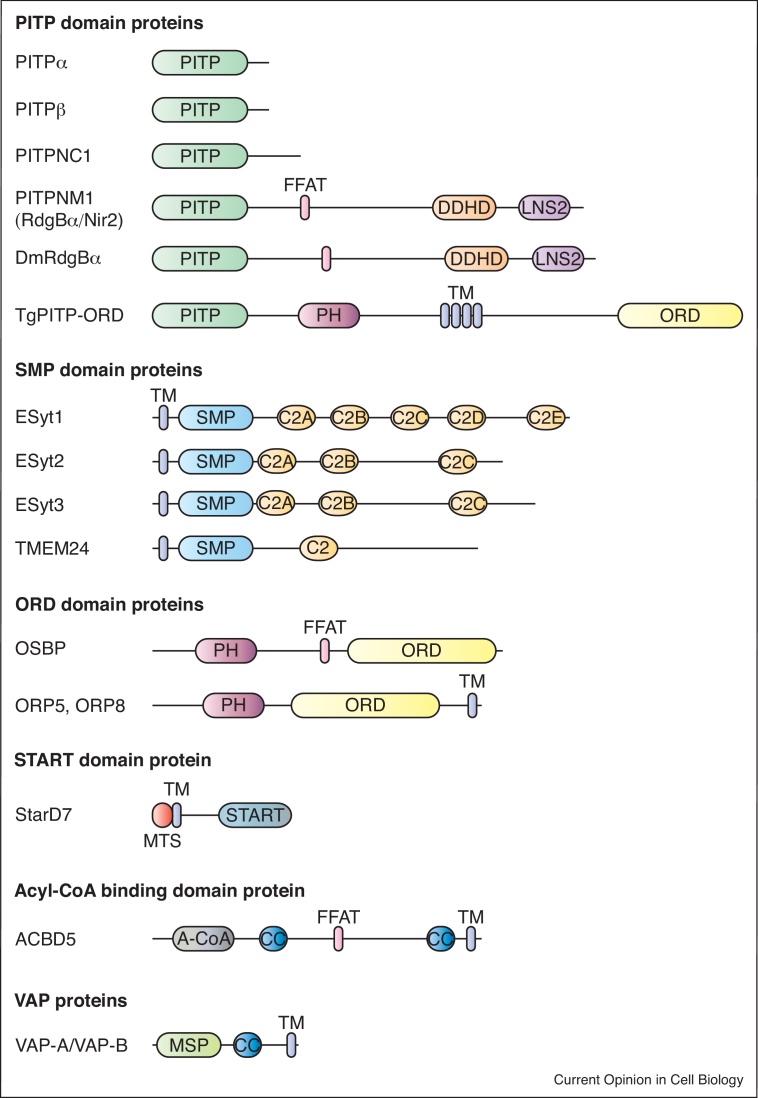
Domain structures of lipid transfer proteins discussed. The PITP domain-containing proteins comprise of five genes encoding the single domain proteins, PITPα and β, which bind and transfer either phosphatidylinositol or phosphatidylcholine. In contrast, PITPNC1 (also known as RdgBβ), also a single domain protein with a disordered C-terminal extension, binds and transfers either PI or PA. This lipid binding and transfer property is shared with the multi-domain PITPNM1 and the *Drosophila* RdgBα. The FFAT motif of PITPNM1/RdgBα binds to the integral ER-localized VAP proteins. Extended synaptotagmins (E-Syts) comprise of a transmembrane domain that localizes the protein to the ER followed by the SMP lipid transfer domain, and multiple C2 domains. OSBP binds and transfers either cholesterol or PI4P facilitating their counter-exchange between the ER and the Golgi. The FFAT motif of OSBP localizes the protein to the ER via binding to VAP. ORP5/ORP8 are integral ER membrane proteins that can associate with the mitochondria by binding to the outer mitochondrial protein, PTP1P5. The ORD domain of ORP5/8 binds PS allowing its transfer to the mitochondria from the ER. Acyl-CoA binding domain containing protein 5 (ACBD5) is a peroxisomal membrane protein with a cytosolic acyl-CoA binding domain. It binds to VAP at the ER due to its FFAT motif. The acyl-CoA binding domain allows for the transfer of very long chain fatty acids from the ER to the peroxisomes. Abbreviations: PITP, phosphatidylinositol transfer protein domain; PH, pleckstrin homology domain; FFAT motif, two phenylalanines in an acidic tract; DDHD domain, domain named after these four conserved residues and may form a metal binding site; LNS2 (Lipin/Ned1/Smp2) domain, found in lipins and lipin homologues from *S. cerevisiae* (Smp2) and *S. pombe* (Ned1); TM, Transmembrane; SMP, synaptotagmin-like mitochondrial lipid binding domain; C2 domain, a structural domain that can bind Ca^2+^ and phospholipids; ORD domain, OSBP-related domain; OSBP, oxysterol binding proteins; ORP, OSBP-related proteins; MTS, mitochondrial targeting sequence; START domain, stAR-related lipid transfer domain; stAR; Steroidogenic acute regulatory protein; A-CoA domain, acyl-CoA binding domain; CC, coiled coil; MSP, Major sperm protein domain; VAP-A/VAP-B, VAMP-associated proteins, A and B.

Phosphatidylinositol transfer proteins (PITPs) were originally identified as soluble factors supporting PLC signaling in mammalian cells [6,7]. The most compelling *in vivo* evidence of their requirement for PI transfer from the ER to the PM comes from studies in *Drosophila* photoreceptors. In fly photoreceptors, the microvillar PM is arranged in close contact with the ER-derived sub-microvillar cisternae (SMC); this is reminiscent of an ER–PM MCS [8]. PLCβ is activated at the microvillar PM, whereas RdgBα, a multi-domain protein with an N-terminal PITP domain (Figure 3), is localized to the SMC. RdgBα mutants show depletion of PM PI(4,5)P_2_ in the resting state and reduced rates of PI(4,5)P_2_ resynthesis during PLC activation [9,10]. These biochemical defects affect photoreceptor function resulting in reduced responses to light and retinal degeneration. The PITP domain of RdgBα can bind and transfer PI *in vitro*, and *in vivo* analyses have shown that RdgBα mutants can be rescued with wild type protein but not with a version that is unable to bind and transfer PI [9]. Conceptually similar results have been shown for the mammalian orthologs of RdgBα, PITPNM1/2 (alt. names: Nir2/Nir3); these studies have been performed in cultured cell-lines with both PITPNM1 (Nir2) and receptors over-expressed [11, 12, 13]. A role for endogenous PITPNM1/2 in supporting endogenous PLC signaling remains to be established; this underscores the need to find mammalian cell types in which the function of endogenous PITPNM proteins and receptors can be studied. Another PITP that supports PLC signaling is PITPα [14^•^,^15^]. Platelets from mouse knockouts in this single domain PITP show reduced PI4P and PI(4,5)P_2_ basal levels; upon stimulation with thrombin, IP_3_ production is minimal and the rise in cytosol Ca^2+^ is reduced [14^•^].

Central to current thinking on the cell biology of lipid transfer at MCS is the idea that LTPs localize to and mediate lipid transfer at these sites. The localization of PITPα to an MCS remains to be determined. However for RdgBα, the protein is detected at the MCS between the SMC and the microvillar PM [16,17]. A recent study demonstrated the importance of this localization. When the PITP domain of RdgBα is delocalized from this interface, *Drosophila* photoreceptors are unable to support PI(4,5)P_2_ resynthesis during high rates of PLC activation [18^••^]. The localization of RdgBα at this MCS is dependent on protein-protein interaction between its FFAT motif and the protein dVAP-A that is enriched at the SMC [18^••^].

In contrast to fly photoreceptors, the MCS in mammalian cells seems dynamically formed during PLC activation and PITPNM1 (Nir2) translocates to these sites to support PM PI(4,5)P_2_ synthesis [12,13,19]. The size of the membrane contact area at MCS is reported to be oblong with the dimensions of ∼120 nm × ∼80 nm in HeLa cells [19]. Multiple signals have been proposed to mediate this PM recruitment including the binding to PA by the LNS2 domain of PITPNM1 [11,20], DAG binding to the DGBL domain of PITPNM1 [13], Ca^2+^ binding to the C2 domain of Extended-Synaptotagmin 1 (E-Syt1) [12] and most recently cortical actin [19]. However, these studies in mammalian cells have only been done where the receptor has been over-expressed suggesting that these PITPNM proteins may only be required during intense stimulation as seen with the *Drosophila* photoreceptors.

### SMP domain proteins

Recent studies have highlighted a new class of LTPs that contain an SMP domain. The extended synaptotagmins (E-Syts) are ER-localized integral membrane proteins comprising an SMP (Synaptotagmin-like mitochondrial lipid binding) domain with multiple C2 domains. The SMP domains dimerize to form a 90 Å cylinder housing two lipids [21,22^••^,^23^]; the SMP domains of E-Syts appear not to have selectivity for any particular glycerolipid. The mammalian E-Syt family has three members: E-Syt1 with 5 C2 domains and E-Syt2/E-Syt3, each with three C2 domains (Figure 3). The C2C domain of E-Syt2 and E-Syt3 form membrane contacts with the PM by binding to PI(4,5)P_2_. In contrast, E-Syt1 translocates to ER–PM junctions after an increase in intracellular Ca^2+^ mediated by its C2A and C2C domain [22^••^,^24^]; Ca^2+^ binding to C2C promotes membrane tethering by C2E binding to PI(4,5)P_2_ at the PM [25^••^]. The lipid transfer activity of E-Syt1 is strictly dependent on the binding of Ca^2+^ to both the C2A and C2C domains. Since E-Syts are only active at elevated cytosol Ca^2+^, lipid transfer can only occur during Ca^2+^ signaling subsequent to PLC activation. Moreover, entry of Ca^2+^ also triggers activation of PLC, and consequently PI(4,5)P_2_ hydrolysis, conditions that would cause E-Syt1 to dissociate from the PM. This raises the question of the relevance of E-Syt1 in replenishing PI(4,5)P_2_ levels at the PM during intense PLC activation. It is notable that mice devoid of all three E-Syts develop normally and are viable and fertile. These animals show upregulation of genes encoding Orp5/8, Orai1, STIM1 and TMEM110, ER–PM MCS proteins that could compensate for loss of E-Syts [26^•^,27^•^].

TMEM24 (C2CD2L) is a protein containing an SMP domain followed by a C2 domain (Figure 3). This SMP domain binds a single PI molecule unlike that of E-Syt2, which can bind two phospholipids. TMEM24 is an ER anchored transmembrane protein that concentrates at ER–PM MCS under resting conditions. TMEM24 binding to the PM is regulated by a phosphorylation cycle mediated by protein kinase C (PKC) and the phosphatase, PP2B both of which are Ca^2+^ dependent enzymes. When phosphorylated by PKC, TMEM24 dissociates from the PM and therefore ceasing transfer; it can only re-associate after dephosphorylation for transfer to resume. Thus TMEM24 maintains basal PI4P and PI(4,5)P_2_ levels; over-expression of TMEM24 in cells leads to increased levels of PI4P and PI(4,5)P_2_ [28^••^]. The TMEM24 protein is highly enriched in pancreatic β-cells and plays a key role in regulating glucose-sensitive insulin release [29].

### PI4P and PI(4,5)P_2_ transfer

Recent studies have identified proteins that can mediate PI(4,5)P_2_ and PI4P removal from the PM. ORP5 and ORP8, localized to ER–PM MCS, can transfer phosphatidylserine (PS) to the PM while removing PI(4,5)P_2_ [30]. RASSF4 was also identified as a regulator of PI(4,5)P_2_ homeostasis by mediating ER–PM junction formation through tethering via E-Syts [31]. ORP5 and ORP8 have previously been implicated in a PI4P/PS exchange cycle that can facilitates PS transfer to the PM coupled to PI4P transfer to the ER [32,33]. Several studies have noted the localization of the PI4P phosphatase Sac1 at ER–PM contact sites depleting PI4P in this microdomain of the ER thus generating a PI4P gradient for this exchange [32, 33, 34, 35].

### PA transfer activity

It is well established that DAG generated by PLC activity is rapidly converted to PA by DAG kinases at the PM [36]. A precursor–product relationship between PA disappearance and new PI synthesis has been demonstrated. Recent studies have shown that the PITP domain of *Drosophila* and mammalian RdgBα/PITPNM1 and PITPNC1 are capable of transporting PA [37,38]. Thus in contrast to PITPα and PITPβ that are PI/PC LTPs, the PITP domains of the RdgB family are PI/PA transfer proteins [38]. Loss of RdgBα in *Drosophila* [9] or PITPNM1 in mammalian cells [13] results in altered PA dynamics during PLC activation. This PI/PA transfer function of RdgBα offers an efficient mechanism for coupling the removal of PA with the supply of PI for PI(4,5)P_2_ resynthesis (Figure 2).

### Diacylglycerol transfer activity

A recent study in mammalian cells depleted of all E-Syts demonstrated sustained accumulation of PM DAG following stimulation by histamine [39,40]. These studies assumed DAG to be derived from PI(4,5)P_2_ hydrolysis. However, DAG at the PM can be derived from both PLC (directly) or phospholipase D (indirectly via PA) activation and both these phospholipases are activated by histamine receptor activation. The accumulation of DAG was rescued by expression of E-Syt1, but not by mutant E-Syt1 lacking the SMP domain. As the SMP domain was found to transfer DAG, this is a potential mechanism for E-Syt1 to regulate the PI(4,5)P_2_ cycle [25^••^].

## Lipid transfer from the ER to other organelles

### ER–Golgi

**Figure 4:**
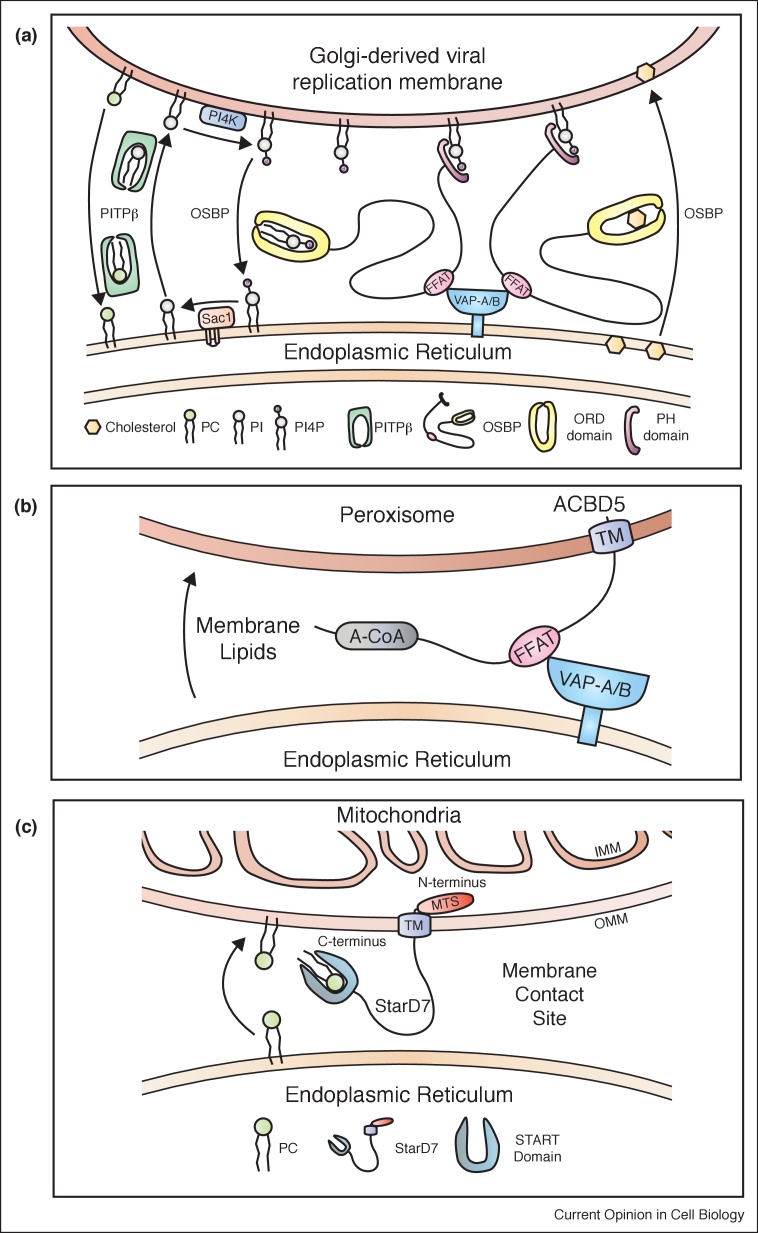
Emerging map of lipid transfer reactions at diverse MCS. (A) Lipid transfer coordinated by OSBP and PITPβ moving cholesterol to Golgi-derived viral replication membranes. PITPβ transfers PI from the ER to the Golgi-derived viral replication membranes where PI4KIIIβ converts it to PI4P. OSBP utilizes the PI4P to co-ordinate the reciprocal transfer of cholesterol to the replication membranes in exchange for PI4P. At the ER, Sac1 dephosphorylates PI4P to PI to maintain the PI4P gradient. (B) Transfer of long chain fatty acids by ACBD5 and ER-peroxisome MCS. (C) Transfer of phosphatidylcholine by STARD7 at ER-mitochondria MCS. Additional tethering complexes will be required to connect the two membranes.

PI synthesized at the ER (Figure 2) is also used for non-PLC dependent processes at other organelle membranes. In this case, PI transfer takes place at MCS using the single domain PITPβ. Oxysterol binding protein (OSBP) is a modular LTP (Figure 3) localized to ER–Golgi contact sites; the FFAT motif permits OSBP to bind VAP at the ER while the PH domain binds to PI4P at the Golgi membrane (Figure 4a). Unlike the ER, the Golgi is enriched in PI4P due to the presence of the PI 4-kinase, IIIβ. The ORD domain drives cholesterol export from the ER to the Golgi through the reciprocal transfer of PI4P from the Golgi to the ER [32,41]. At the ER, Sac1 converts PI4P into PI. Since PI synthesis is restricted to the ER, a supply of PI to the Golgi for PI4P synthesis is necessary. PITPβ is localized to the Golgi and a previous study reported that its depletion led to decreased PI4P levels and disrupted COP1 mediated retrograde transport from the Golgi to the ER [42]. Thus PITPβ is ideally localized for maintaining Golgi PI4P levels that is consumed during cholesterol transfer. Evidence to support this comes from recent studies which has identified PITPβ as a host factor required for positive-strand RNA viral replication [43,44]. For replication, the virus builds a membrane-associated replication complex which is Golgi-derived that is tightly associated with the ER. Interestingly OSBP, PI4KIIIβ and Sac1 all of which are also localized at this MCS, are required for both PI4P homeostasis and viral replication [43,44] (Figure 4a). What recruits PITPβ to these MCS is unclear. In *Toxoplasma gondii*, a multi-domain protein of 1912 a.a. incorporates a PITP domain with a PH domain, 4 transmembrane domains and an oxysterol binding domain (PITP-PH-4TM-OSBP) (Figure 3). Thus the concept that a PITP domain works in concert with an OSBP domain protein appears to be amalgamated into a single protein in some organisms.

### ER–peroxisomes

Peroxisomes depend on the ER for their lipid composition and the ER receives lipid precursors for plasmalogen biosynthesis (ether phospholipids) from peroxisomes. The tether that links these two organelles is the ER protein VAP-B interacting via its MSP domain with the FFAT-like motif of acyl-CoA binding domain-containing 5 (ACBD5), a peroxisomal tail-anchored membrane protein [45^••^,46^••^,^47^] (Figure 4b). An intact VAP-ACBD5 tether is required for peroxisome growth, plasmalogen synthesis and maintenance of cellular cholesterol levels [45^••^]. The ACB domain of ACBD5 preferentially binds very long chain fatty acyl-CoAs and transfers them to peroxisomes. Mutations in ACBD5 show elevated levels of very long chain fatty acids and a defect in peroxisomal β-oxidation of very long chain fatty acids [48]; patients with ACBD5 deficiency manifest with retinal dystrophy [49].

### ER–autophagosome

The transfer of PI from the ER is also required for autophagosome biogenesis [50]. PI is synthesized in the ER from CDP-DAG by PI synthase (PIS) (Figure 2). Over-expressed PIS localizes to a highly dynamic compartment of the ER and at leading edges of tubules [51,52]. Early autophagic structures are formed in close apposition to the ER and recent studies reveal that autophagosome formation requires a subdomain of the ER, which is highly enriched in PIS. The ULK complex first localizes to the PIS-enriched ER subdomain and then translocates to the ATG9A-positive autophagosome precursors in a PI3P-dependent manner. PI in the PIS-enriched membrane is required for autophagosome formation. LTPs that could transfer PI from the PIS-enriched subdomain to the ATG9A vesicles [50,53] remain to be identified.

### ER–mitochondria

Mitochondria can synthesize PA, PG, cardiolipin and PE. However, PC, PI and PS have to be imported from the ER. PS imported into mitochondria is used by PS decarboxylase to produce PE at the inner mitochondrial membrane. Close contacts between the ER and mitochondria are important for lipid transfer; ER to mitochondria PS transfer slows down significantly in yeast cells missing both the ER-shaping reticulon proteins and the ERMES complex. This defect in PS transfer could be corrected by expression of a protein that artificially tethers the ER and mitochondria [54]; these findings have now been extended to mammalian cells [55]. The ER that associates with mitochondria is enriched in PS synthase [56,57]. Recent studies have identified ORP5/ORP8 (Figure 3) to localize to ER–mitochondria contacts, interact with the outer mitochondrial protein, PTP1P5 and transfer PS from the ER to mitochondria [58^••^]. Depletion of ORP5/ORP8 leads to altered mitochondrial morphology and function. Together, these findings indicate that PS production and transport at the ER–mitochondrial MCS is required to support mitochondrial function.

PC is the dominant lipid of mitochondria and recent studies emphasize StarD7, a member of the START family facilitates PC transfer from the ER to the outer mitochondrial membrane (OMM). StarD7-I, the longer isoform, contains a mitochondrial targeting sequence followed by a transmembrane domain anchoring the protein to the OMM [59] (Figure 3). The C-terminal START domain would then extend into the cytoplasm and shuttle PC from the ER to OMM at the ER–mitochondria contact sites [60^••^] (Figure 4c). Loss of StarD7 results in embyronic lethality and compromised mitochondrial function [61^••^]. Interestingly, loss of StarD7 results in only a partial loss of PC in mitochondria and suggests that other PC transfer proteins such as PCTP (StarD2) and StarD10 may have a role in PC transfer.

## General conclusions

LTPs were initially identified as soluble single domain proteins that could transfer lipids between membrane compartments *in vitro*. However, in recent years, it has emerged that such lipid transfer domains occur in a diverse range of proteins in conjunction with other protein domains that in themselves appear to have no LTP activity. One emerging function of these additional domains is their ability to act as protein targeting signals thus ensuring the positioning of lipid transfer activity at specific and in some cases unique locations with cells. One such location that has emerged are MCS between the ER and multiple cellular organelles where LTPs seem to localize suggesting their ability to transfer lipid locally at these subcellular locations.

Numerous cell biological studies have informed on the localization and biochemical activity of LTPs in cultured cells. However, most are performed with the LTP overexpressed; while this has provided initial insights, it will be essential that going forward the localization of the endogenous LTPs in cells be established. Such studies will help to identify *in vivo* cell types where a given LTP is enriched leading to the development of model cell types where the function and regulation of endogenous LTPs can be studied.

In contrast to cell biological studies, there have been limited analyses of the role of LTPs in physiological processes *in vivo*. Where they have been done, in many cases, phenotypes have been surprisingly limited. This observation may reflect functional redundancy between multiple LTPs that can perform the same biochemical activity; this possibility is reflected in the finding that multiple genes encoding an LTP are found in mammalian genomes. Studies to address this redundancy will be important to understand the overall contribution of this class of molecules in regulating lipid homeostasis in cell physiology.

## Conflict of interest statement

Nothing declared.

## References and recommended reading

Papers of particular interest, published within the period of review, have been highlighted as:

• of special interest
•• of outstanding interest
